# Cepharanthine: a review of the antiviral potential of a Japanese-approved alopecia drug in COVID-19

**DOI:** 10.1007/s43440-020-00132-z

**Published:** 2020-07-22

**Authors:** Moshe Rogosnitzky, Paul Okediji, Igor Koman

**Affiliations:** 1 MedInsight Research Institute, Pekeris 4, Weizmann Science Park, 7670204 Rehovot, Israel; 2 Institute for Personalized and Translational Medicine, Ariel University, 40700 Ariel, Israel

**Keywords:** Cepharanthine, COVID-19, Antiviral, Coronavirus, SARS-CoV-2

## Abstract

Cepharanthine (CEP) is a naturally occurring alkaloid derived from *Stephania cepharantha* Hayata and demonstrated to have unique anti-inflammatory, antioxidative, immunomodulating, antiparasitic, and antiviral properties. Its therapeutic potential as an antiviral agent has never been more important than in combating COVID-19 caused by severe acute respiratory syndrome coronavirus type 2 (SARS-CoV-2) virus. Cepharanthine suppresses nuclear factor-kappa B (NF-κB) activation, lipid peroxidation, nitric oxide (NO) production, cytokine production, and expression of cyclooxygenase; all of which are crucial to viral replication and inflammatory response. Against SARS-CoV-2 and homologous viruses, CEP predominantly inhibits viral entry and replication at low doses; and was recently identified as the most potent coronavirus inhibitor among 2406 clinically approved drug repurposing candidates in a preclinical model. This review critically analyzes and consolidates available evidence establishing CEP’s potential therapeutic importance as a drug of choice in managing COVID-19 cases.

## Introduction

The emergence of the 2019 novel coronavirus disease (labelled COVID-19) in December 2019 triggered an unprecedented global public health challenge. The disease is a clinical syndrome caused by the severe acute respiratory syndrome coronavirus type 2 (SARS-CoV-2) virus. With millions of cases globally, COVID-19 has become the world’s largest and deadliest infectious disease outbreak since the Spanish flu of the early 1900s and it represents a serious public health problem that requires urgent solutions [1, 2]. So far, there is no confirmed cure for the disease which has led to hundreds of thousands of mortalities. The high transmission and exponential growth rate of the SARS-CoV-2 virus coupled with the slow process of developing vaccines and custom-designed pharmacological treatments have pointed at the need to quickly repurpose existing drugs such as cepharanthine (CEP) as potential treatments for COVID-19.

Cepharanthine is an alkaloid that has been used in Japanese medicine since 1951 primarily to treat radiation-induced leukopenia, alopecia areata, and alopecia pityrodes [3] as well as exudative middle-ear catarrh, and viper bite. It has been shown to possess anti-inflammatory, anti-oxidative, immuno-modulating, anti-parasitic, and antiviral properties which suggest possible use in a viral disease such as COVID-19 [3, 4]. More specifically, the antiviral effects of CEP have been demonstrated against HCoV-OC43, which is a mildly pathogenic human coronavirus [5], and severe acute respiratory syndrome coronavirus (SARS-CoV), which was responsible for the 2003 severe acute respiratory syndrome (SARS) outbreak [6]. Additionally, CEP was recently identified as the most effective drug against SARS-CoV-2-related pangolin coronavirus, a less pathogenic model for SARS-CoV-2, in a large drug screen of 2406 clinically approved drugs [7]. In the light of these findings and the close homology between the genome sequences of SARS-CoV and SARS-CoV-2 [2], CEP has become a drug of interest for treating COVID-19.

Extraordinary efforts are underway globally to identify safe and effective treatments for COVID-19. However, despite these efforts, only remdesivir currently has the United States Food and Drug Administration’s (FDA) emergency use authorization for the treatment of COVID-19, and there is still limited information about the safety and effectiveness of this drug in clinical use [8, 9]. Chloroquine and hydroxychloroquine, which had earlier been granted FDA emergency use authorization in COVID-19, saw this authorization revoked due to lack of efficacy [10]. This further bolsters the need to critically examine and test the role of CEP as a potential anti-COVID-19 medication. This review is aimed at expediting this process by analyzing and consolidating published evidence on the antiviral effects of CEP and its therapeutic potential as a possible treatment for COVID-19.

### A brief overview and current uses of cepharanthine

**Fig. 1:**
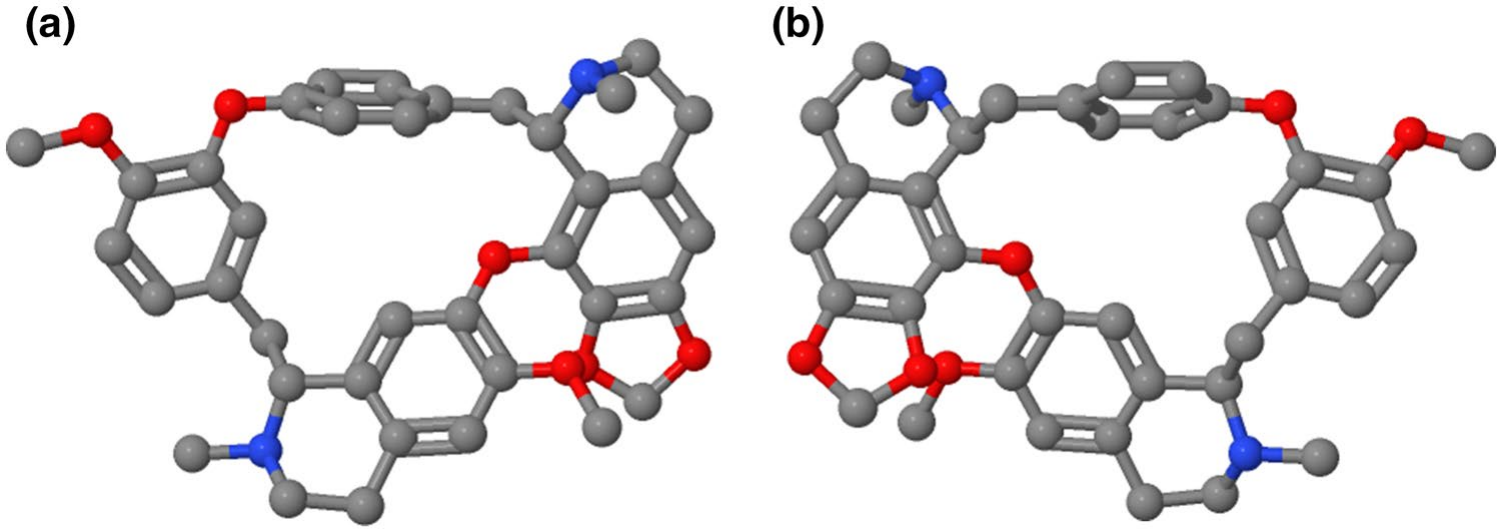
**a**, **b** Chemical structure of cepharanthine; front view (**a**) and back view (**b**)

Cepharanthine is a natural alkaloid derived from plants of the genus Stephania native to Taiwan, China, Cambodia, Laos, Vietnam, and a few other southeastern Asian countries. Over the last 70–80 years, several members of the species have been used in Asia as traditional remedies for fever [4]. More specifically, *Stephania rotunda* Lour and *Stephania cepharantha* Hayata have been the most common species used as herbal medications in the region. There has been increasing interest in CEP because of its unique 1-benzylisoquinoline moiety (Fig. 1a, b), similarities with natural polypeptides, physiological properties, and long-established excellent safety profile [11].

Cepharanthine was first purified in 1934 by a Japanese pharmacist and named after one of its key sources: *Stephania cepharantha* [4]. Chemically, CEP is classified as a biscoclaurine alkaloid and is a member of the family of bisbenzylisoquinoline cyclic alkaloids which also includes tetandrine, daphnoline, berbamine, and several others [4]. In its chemical structure, CEP has two benzylisoquinoline units attached head-to-head which gives it an elliptic macrocyclic structure and confers it with its unique chemical properties such as its ether solubility, optical activity, and ability to reduce the fluidity of a wide variety of biological membranes [4, 12].

There have been several unsuccessful attempts at synthesizing CEP in a cost-effective manner [3]. So far, CEP has been optimally extracted using different patented approaches and formulated into orally administered tablets and powder, as well as injectables [4]. It is an approved medication indicated for managing radiation-induced leukopenia [13], alopecia areata [4], alopecia pityrodes [3], idiopathic thrombocytopenic purpura [14], middle ear catarrh, and bites from vipers and other venomous snakes [3]. At present, there is no clear consensus on the optimal dosing of CEP although doses of 2–60 mg per day have been used safely and effectively for the management of a wide variety of conditions [3]. Available data show that CEP has a half-life of 31.3–36.9 h with steady state levels achieved after five to six repeated dosings of 100 mg/day and a maximum of 9.6% of unchanged drug is left following oral dosing [15]. The time to maximum serum concentration after oral administration of a single 10 mg to 60 mg dose in healthy adult males ranges between 1.1 and 2.5 h [3]. Following absorption, CEP is metabolized extensively in the liver and distributed to the tissues, where it binds strongly.

Broad pharmacologic effects of CEP have been demonstrated in vitro and in vivo using various experimental models. Ershun et al. demonstrated CEP’s anti-inflammatory properties using an in vivo mouse model of mastitis and showing that CEP reduced the levels of tumor necrosis factor α (TNF-α), interleukin-1β (IL-1β), and interleukin-6 (IL-6) through an attenuation of the natural inflammatory response [16]. Similarly, an experiment using a mouse model of acute lung injury and lipopolysaccharide (LPS)-stimulated RAW264.7 cells showed that CEP does suppress nuclear factor-kappa B (NF-κB) activation, lipid peroxidation, nitric oxide (NO) production, and expression of cyclooxygenase; thereby causing a dampened inflammatory response and subsequent inhibition of vascular smooth muscle cell proliferation and migration associated with atherosclerosis [17].

Also, CEP has been shown through both in vitro and in vivo experiments to prevent cell death by suppressing endotoxin-induced production of NO in macrophages; thereby protecting against widespread endotoxin damage in septic shock [18]. Other key effects of CEP include in vivo inhibition of superoxide generation and luminol-dependent chemiluminescence in guinea-pig neutrophils [19], in vitro stimulation of autophagy and autophagic cell death in a panel of apoptosis-resistant cancer cells [20], in vivo and in vitro inhibition of vascular endothelial growth factor and IL-8 (which are key pro-angiogenic substances) and NF-κB activity [21]. Cepharanthine is also known to block *Plasmodium falciparum* development in its ring stages [22] and potentiate anti-cancer activity of chemotherapeutic agents by inhibiting drug transporters leading to the accumulation of the agents in cells [23].

### Antiviral effects

Beyond the general pharmacological effects identified and discussed earlier, there have been in vitro studies pointing at specific antiviral properties of CEP that make it a potential drug of choice in the management of COVID-19 (Table 1).

**Table 1:** Summary of studies examining antiviral effects of cepharanthine

Virus	Cell type	Antiviral effect	Mechanism of action
HIV-1 [24]	U1 monocytes and ACH-2 T lymphocytes	Inhibits viral replication in U1 monocytic cells, but not in ACH-2 lymphocytic cells	Inhibits NF-κB activation
HTLV-1 [28]	MT-2 cells^a^ SIT cells^b^	Inhibits the proliferation of both cell lines; and induces apoptosis of SIT cells	Inhibits NF-κB activation
HBV [30]	HepG2 cells^c^	Inhibits viral replication and suppresses viral HBeAg antigen production	Suppresses HBV via downregulation of host Hsc70^d^ expression
SARS-CoV [6]	VeroE6^e^	Inhibits viral replication	Mechanism unknown
HCoV-OC43 [5]	MRC-5 fibroblasts	Inhibits viral replication and infectivity; and dampens virus-induced host response	Blocks expression of the viral spike protein and nucleoprotein
SARS-CoV-2 [1]	VeroE6/TMPRSS2	Inhibits viral entry and replication	Binds to the spike protein and interferes with viral engagement to ACE2
SARS-CoV-2 [34]	Vero cells	Suppresses viral infectivity	Mechanism unknown
SARS-CoV-2 [35]	Calu-3 human lung cells	Inhibits viral infectivity	Mechanism unknown
GX_P2V^f^ [7]	VeroE6^e^	Inhibits viral replication and cytopathic effects	Mechanism unknown

^a^MT-2 is an HTLV-1-transformed T-cell line

^b^SIT is a leukemia cell line established from patients with adult T-cell leukemia

^c^Human hepatoma cell line—HepG2

^d^Heat stress cognate-70 is a host protein critical to HBV replication

^e^Monkey kidney epithelial cell line VeroE6

^f^Pangolin coronavirus GX_P2V/pangolin/2017/Guangxi

### In vitro* effects of CEP on Human Immunodeficiency Virus (HIV)*

**Fig. 2:**
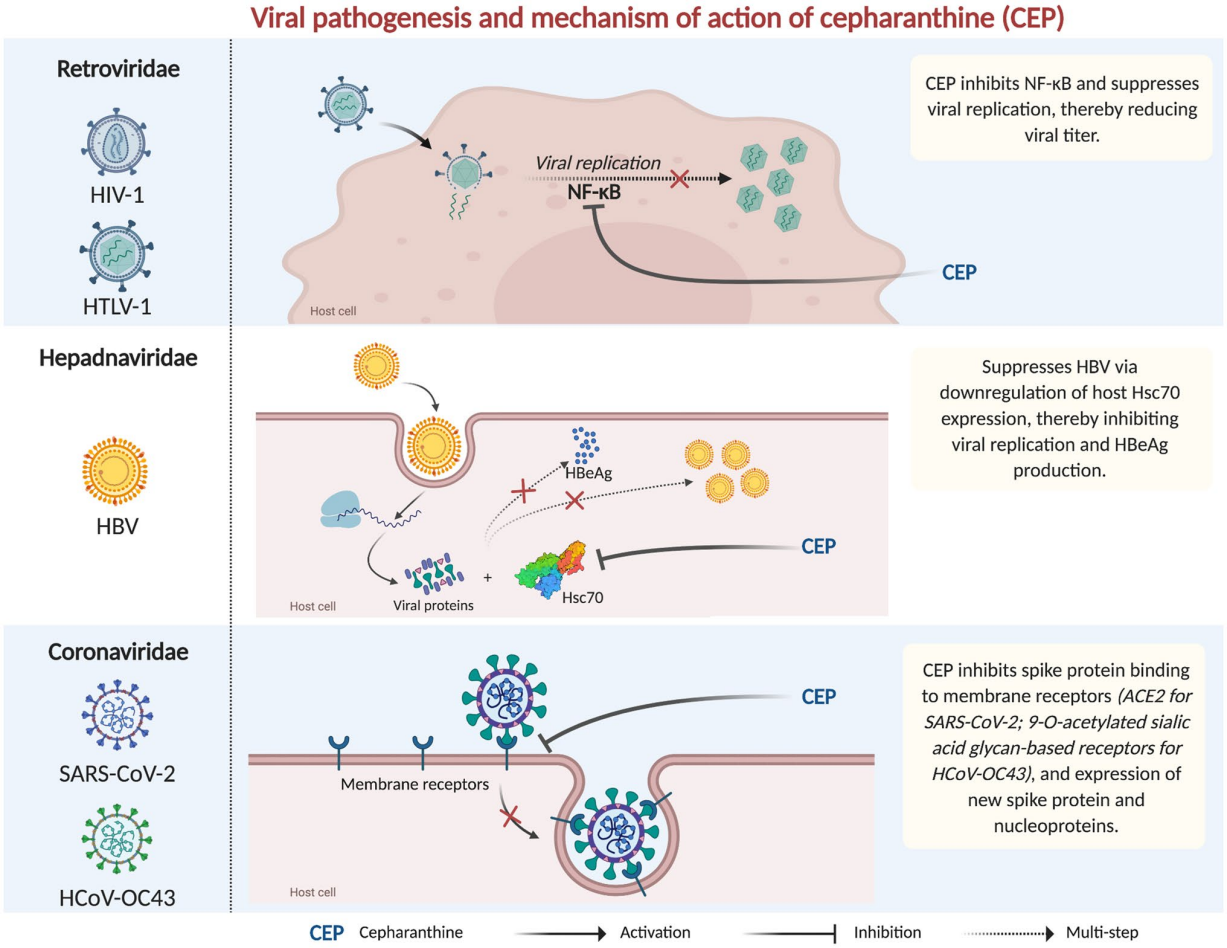
Pathogenesis of HIV-1, HTLV-1, HBV, SARS-CoV-2, and HCOV-OC43; and mechanism of action of cepharanthine against them

Cepharanthine has been demonstrated to have unique antiviral properties on the human immunodeficiency virus (HIV). Okamoto et al. showed the extent to which CEP inhibits replication of HIV type 1 (HIV-1) in infected monocytic and lymphocytic cell lines [24]. At a 50% effective concentration (EC_50_) of 0.016 µg/ml and a cytotoxic concentration (CC_50_) of 2.2 µg/ml, the researchers found CEP to dose-dependently inhibit the replication of the virus in TNF-α-stimulated U1 monocytic cells. This effect was attributed to CEP's ability to suppress HIV-1 long terminal repeat-driven gene expression by inhibiting the activation of NF-κB (Fig. 2). Of all the cellular factors involved in HIV-1 replication and expression, NF-κB is the most potent activator of its expression [25], and its inhibition by CEP goes a long way in dampening the virus’s pathogenicity.

Additional in vitro experiments have gone further to compare CEP’s anti-HIV-1 abilities with other alkaloid derivatives of cepharanoline. According to Baba et al. CEP was found to be the most active inhibitor of NF-κB (among 96 derivatives of cepharanoline) and thus, the most potent agent against HIV-1 activity in chronically infected cells [26]. The ability of CEP to easily penetrate the central nervous system (CNS) makes it even more useful in managing HIV-1-associated conditions of the CNS [27]. Compared with conventional antiviral agents currently used in the management of HIV (such as reverse transcriptase and protease inhibitors), CEP appears to be better suited to serve as both a prophylactic and therapeutic agent in preventing and treating HIV-associated CNS disorders, primarily because it easily traverses the blood–brain barrier and can suppress the production of inflammatory cytokines that are responsible for CNS damage [24].

### In vitro* effects of CEP on human T-lymphocytic virus (HTLV)*

Similar to the effects of CEP on HIV-1, the human T-lymphotropic virus type 1 (HTLV-1), which is the causative agent of adult T-cell leukemia, has been found to be susceptible to CEP. In vitro experiments by Toyama et al. show that the use of CEP alone or in combination with tetrahydrotetramethylnaphthalene (TMNAA) derivative triggered apoptosis of HTLV-1 infected cells through the caspase-dependent pathway [28]. The authors postulated that this effect may be due to the inhibition of the NF-κB signalling pathway found to be upregulated in infected cells (Fig. 2). The combination of CEP with TMNAA in this study was observed to generate synergistic antiproliferative activity which suggests that the dose of either agent can be reduced to achieve the same or even better efficacy than the use of one agent alone. Furthermore, there is evidence pointing at the ability of CEP to reduce or reverse the resistance of cancer cells to antitumor agents, suggesting that combination chemotherapy with CEP has superior benefits [5, 28].

### In vitro* effects of CEP on hepatitis B virus (HBV)*

Lamivudine is a nucleoside analogue commonly used as an antiviral agent against HBV. Over time, resistance against lamivudine has been on the rise with as much as 76% of patients developing drug resistance after 5 years of lamivudine therapy [29]. The need for alternatives has never been more apparent and CEP has proven useful in addressing this challenge. In a study comparing the antiviral effects of lamivudine, CEP, and adefovir on virus antigen production and HBV replication; lamivudine was found to have the most efficacy of all three substances when tested on wild-type HBV [30]. This was followed by CEP which had a higher rate of inhibition of both virus replication and viral antigen production than adefovir on both wild-type and lamivudine-resistant HBV subtypes. When tested on HepG2 cells, both CEP and adefovir exhibited similar cytotoxic effects.

### In vitro* effects of CEP on SARS-CoV and HCoV-OC43*

Severe acute respiratory syndrome is a highly contagious atypical respiratory illness caused by the human coronavirus: SARS-CoV. Responsible for over 8000 deaths between 2002 and 2003, scientists have been working hard to find a cure [31]. Cepharanthine is an agent that has shown preclinical promise in the treatment of SARS. Zhang et al. conducted in vitro experiments to assess the extent of anti-SARS-CoV activity using VeroE6 cells infected with SARS-CoV [6]. The researchers split the cells into four groups, where cells in the first group were pre-treated with CEP prior to infection and cells in the second group were treated with CEP post-infection with the virus. The third group had cells co-treated with CEP and the virus. The fourth group of cells were also co-treated with CEP and the virus but the mixture of CEP and virus were incubated at 37 °C for 2 h prior to application on the VeroE6 cells. Following treatment, viral cytopathic effect was inhibited by a CEP concentration of 10 µg/ml across all groups, with the 50% inhibitory concentration (IC_50_) ranging between 6.0 and 9.5 µg/ml for the four treatments [6].

Even though Zhang et al. [6] had no clear explanation for their findings, their results were not totally surprising as CEP has also been demonstrated to inhibit similar human coronaviruses such as the human coronavirus type OC43 (HCoV-OC43). A 2019 study examined the effects of bisbenzylisoquinoline alkaloids (including CEP) on human MRC-5 lung cells infected with HCoV-OC43 [5]. Following pretreatment with CEP and HCoV-OC43, MRC-5 cells that were exposed to CEP prior to the viral inoculation showed no virus-induced cytotoxicity or morphological changes with the CC_50_ and EC_50_ found to be 10.54 µmol/L and 729.7 nmol/L, respectively. Another key finding from the study was the increase in the viability of infected cells in a dose-dependent manner [5]. These findings were attributed to the ability of CEP to inhibit viral RNA replication, block expression of viral proteins, and suppress production of proinflammatory molecules towards preventing an exacerbated cytokine response to the viral infection.

These findings are crucial to understanding the potential of CEP as an efficacious treatment option for COVID-19 particularly because of the similarities between HCoV-OC43, SARS-CoV, and SARS-CoV-2; all of which are human coronaviruses [32]. As pointed out previously, SARS-CoV-2 shares 79.6% gene sequence homology with SARS-CoV and both viruses utilize the same cell-membrane receptor—angiotensin converting enzyme 2 (ACE2)—to achieve viral entry [2] (Fig. 2). Unlike SARS-CoV and SARS-CoV-2, however, HCoV-OC43 uses 9-*O*-acetylated sialic acid glycan-based receptors to mediate viral entry [5]. Nonetheless, given the remarkable effect of CEP on viral binding, entry, and replication in the case of both HCoV-OC43 and SARS-CoV; there is a probability that CEP will show similar effects on the newly discovered SARS-CoV-2.

### *Effects of CEP on SARS-CoV-2 (*in vitro* and predictive models)*

Recent studies indicate that CEP could be an effective treatment against SARS-CoV-2 with minimal toxicity. A group of researchers recently conducted a panel screening of drugs (including nelfinavir, CEP, and chloroquine) already approved for the treatment of various conditions, on an in vitro SARS-CoV-2 cell culture model [1]. Using VeroE6/TMPRSS2 cells inoculated with SARS-CoV-2, nelfinavir and CEP were found to have excellent antiviral effects on SARS-CoV-2 either singly or in combination. Nelfinavir is a known protease inhibitor and the modeling data from the study confirmed that its ability to inhibit SARS-CoV-2 replication was due to inhibition of the virus’ main protease. CEP, on the other hand, was found to predominantly block viral entry by interfering with the ability of the virus to attach to its target cell via its S glycoprotein [33]. When combined, both agents work synergistically to target both the viral entry and replication phases of SARS-CoV-2 infection, suggesting superior outcomes when compared with chloroquine and lopinavir which are among the currently used drugs for COVID-19 treatment [1]. Additionally, the EC_50_ and CC_50_ ratio in this combination was over 70 (IC_50_ 0.35 µmol/L, CC_50_ 25.10 µmol/L) implying that SARS-CoV-2 inhibition can be achieved with minimal toxicity.

In yet another drug screening to repurpose already approved drugs into possible COVID-19 treatments, Fan et al. tested over 2400 drugs on VeroE6 cells infected with a 2019-nCoV-related pangolin coronavirus (GX_P2V/pangolin/2017/Guangxi) as an alternative to SARS-CoV-2 because of its low human pathogenicity [7]. The spike protein of the pangolin coronavirus has 92.2% amino acid sequence similarity with the spike protein of the clinical isolate of SARS-CoV-2, thereby supporting its use as a model for SARS-CoV-2. Of the 2406 drugs tested; CEP, selamectin, and mefloquine hydrochloride were the only three drugs observed to completely inhibit the virus’ cytopathic effect. Of these three drugs, CEP had the most potent antiviral activity even at low IC_50_ (0.73 μmol/L) and CC_50_ (39.30 μmol/L); similar to what had been reported in tests against SARS-CoV and HCoV-OC43 [5, 6]. Also, CEP was found to significantly decrease viral RNA yields across the entry and post-entry phases by as much as 2.17 fold and 1618 folds, respectively, relative to normal infections without any treatment [7]. This implies that CEP plays inhibitory roles both at the entry and post-entry phases of SARS-CoV-2 infection.

The antiviral potential of CEP against SARS-CoV-2 has been further demonstrated in recent in vitro studies using SARS-CoV-2 models. Jeon et al. who screened 3000 approved drugs for anti-SARS-CoV-2 activity, shortlisted CEP as one of 24 drugs that demonstrated antiviral activity against SARS-CoV-2 in Vero cells [34]. In a follow-up study by the same group utilizing Calu-3 cells, CEP ranked in fifth place with an IC_50_ of 30 µM (as opposed to an IC_50_ of 4.47 µM in Vero cells) [35].

Ruan et al. indicated that the antiviral activity of CEP against SARS-CoV-2 is due to its ability to combine well with a multi-subunit complex of nonstructural proteins (NSP) [36]. This conclusion was based on CEP’s ability to combine well with the NSP12-NSP7-NSP8 complex in a virtual screening based on homologous models of SARS NSPs. Being an RNA virus, the NSP12-NSP7-NSP8 complex in SARS-CoV-2 is deemed critical for viral replication and transcription [37]. By binding to this NSP machinery, CEP blocks the ability of SARS-CoV-2 to replicate and produce relevant proteins. The inhibition of the Niemann-Pick type C disease-causing gene (NPC1) is another mechanism through which CEP has been shown to impair the infectivity of SARS-CoV-2 and subsequent replication [38]. As a NPC1 inhibitor, CEP causes disruption of cellular/lysosomal lipid homeostasis and potentially inhibits the entry, replication, and exit of SARS-CoV-2 [38, 39].

So far, encouraging preclinical evidence about CEP's safety profile, its availability, and unique antiviral properties especially against specific beta-coronaviruses supports its consideration as a drug of choice in the treatment of human coronavirus diseases. In vivo testing and clinical trials are urgently required to provide additional data supporting the development of CEP into a COVID-19 treatment, spurred by its superior antiviral and antiinflammatory activities in preclinical settings relative to established antiviral agents. The ability of CEP to disable the attachment and entry of SARS-CoV-2 into target cells demonstrates its great therapeutic potential as a drug to combat COVID-19. Also, its low EC_50_ and CC_50_ achievable with oral dosing, and synergistic properties when combined with other antiviral medications (such as nelfinavir and mefloquine), further supports its excellent pharmacokinetic profile and establishes its importance as a candidate in the fight against COVID-19.

## Conclusion

In summary, CEP has been demonstrated pre-clinically to have significant antiviral effects against viruses such as HIV, HTLV, HBV, SARS-CoV, and now, SARS-CoV-2, which is the culprit behind the world’s largest and deadliest infectious disease outbreak within the last 100 years. Monotherapy with CEP has been demonstrated to inhibit viral entry and attenuate the potential inflammatory effects that may result from viral infection. These effects can be accentuated through combinations of CEP with other antiviral agents such as nelfinavir, mefloquine, or selamectin. Considering the fact that the majority of available literature on the antiviral effects of CEP have been from in vitro studies, further research on in vivo models and clinical trials are urgently called for to confirm the usefulness of CEP in real-life COVID-19 cases and enable the full maximization of the potentials of this naturally-occurring alkaloid. Given the well-established safety profile and clear antiviral and antiinflammatory effects of CEP, it presents as an optimal candidate for rapid repurposing to combat viral infection from SARS-CoV-2 and its COVID-19 sequelae.

## Abbreviations

ACE2: Angiotensin converting enzyme 2
CC_50_: 50% Cytotoxic concentration
CEP: Cepharanthine
CNS: Central nervous system
COVID-19: 2019 Novel coronavirus disease
EC_50_: 50% Effective concentration
FDA: Food and drug administration
HBV: Hepatitis B virus
HCoV-OC43: Human coronavirus type OC43
HIV: Human immunodeficiency virus
HTLV: Human T-lymphocytic virus
IC_50_: 50% Inhibitory concentration
IL-1β: Interleukin-1β
IL-6: Interleukin-6
LPS: Lipopolysaccharide
NF-κB: Nuclear factor-kappa B
NO: Nitric oxide
NPC1: Niemann-pick type C disease-causing gene
NSP: Non-structural protein
SARS: Severe acute respiratory syndrome
SARS-CoV: Severe acute respiratory syndrome coronavirus
SARS-CoV-2: Severe acute respiratory syndrome coronavirus type 2 virus
TMNAA: Tetrahydrotetramethylnaphthalene
TNF-α: Tumor necrosis factor α
